# Prevalence of asymptomatic *Plasmodium* species infection and associated factors among pregnant women attending antenatal care at Fendeka town health facilities, Jawi District, North west Ethiopia: A cross-sectional study

**DOI:** 10.1371/journal.pone.0231477

**Published:** 2020-04-21

**Authors:** Adane Tilahun, Mulat Yimer, Woynshet Gelaye, Banchamlak Tegegne

**Affiliations:** 1 Debre-Markos Referral Hospital, Medical Laboratory Service, Debre-Markos, Ethiopia; 2 Department of Medical Laboratory Sciences, School of Health Sciences, College of Medicine and Health Sciences, Bahir Dar University, Bahir Dar, Ethiopia; 3 Amhara Public Health Institute, Bahir Dar, Ethiopia; Instituto Rene Rachou, BRAZIL

## Abstract

**Background:**

Malaria in pregnancy remains a major public health problem especially in sub-Saharan Africa. In malaria endemic areas, majority of pregnant women may remain asymptomatic but still associated with complications on the mother and her foetus. They also serve as reservoirs and act as transmitters of infection. Despite these effects, the prevalence of asymptomatic *Plasmodium* species infections among pregnant women attending antenatal care has not been yet studied at the study area. Therefore, the aim of this study was to assess the prevalence of asymptomatic *Plasmodium* species infections among pregnant women attending antenatal care at Fendeka town health facilities.

**Methods:**

Health facility based cross -sectional study was conducted from February to March 2019. A total of 331 participants were enrolled by using convenient sampling technique. Socio-demographic and associated factors were collected by a face to face interview. All the 331 samples were tested using rapid diagnostic tests (RDTs) and microscopy. However, only 83 dried blood spot (DBS) samples out of 331 participants, were collected by using systematic random sampling technique for molecular analysis. Data was analysed using SPSS version 20. Descriptive statistics were used to determine the prevalence of asymptomatic *Plasmodium* species infections. Univariate logistic regression was employed to assess factors associated with asymptomatic *Plasmodium* species infection. Variables with P-value < 0.25 in the univariate logistic regression were selected for multivariate logistic regression analysis model. Odds ratios with 95% confidence intervals were calculated and P- values < 0.05 were considered as statistically significant.

**Results:**

Overall, 37 (11.2%) asymptomatic *Plasmodium* species infections were detected using: RDTs, microscopy and real-time PCR altogether. The asymptomatic *Plasmodium* species infection prevalence was 17 (5.1%), 30 (9.1%) and 15(18.1%) using RDTs, microscopy and real-time PCR, respectively. Asymptomatic *Plasmodium* species infections were more likely to occur in primigravida (AOR: 4.51, 95% CI: 1.27–16.03), secundigravida (AOR: 3.87, 95% CI: 1.16–12.93), rural inhabitants (AOR: 4.51, 95% CI: 1.72–11.84) and in participants who did not use indoor residual spray (IRS) for the last one year (AOR: 3.13, 95% CI: 1.47–6.66).

**Conclusions:**

The prevalence of asymptomatic *Plasmodium* species infection was 11.2%. Pregnant women who reside in the rural area, primigravidae, secugravidae and those who did not utilize indoor residual spray for the last one year were at high risk of infection. Therefore, routine laboratory diagnosis of asymptomatic *Plasmodium* species infection among pregnant women should be adopted as a part of the antenatal care.

## Background

Malaria is a serious public health problem in tropical and subtropical regions of the world and caused by five *Plasmodium* species [1, 2]. Of these species; *P*. *falciparum* and *P*. *vivax* pose the greatest threat in the world. The disease is transmitted to people mainly via the bites of infected female *Anopheles mosquitoes* [2]. The Ethiopian Ministry of Health (MoH) reported that 68% of the country’s land mass is favourable for malaria transmission [3]. Sixty percent and 40% of the malaria cases are caused by P. *falciparum* and *P*. *vivax*, respectively [4]. On the contrary, the dominance shifting from *P*. *falciparum* to *P*. *vivax* in highland areas was reported by Gemeda et al [5].

Globally, 125 million pregnant women are at risk of getting malaria every year. Of these, most cases and deaths are in Sub-Saharan Africa [1]. In Ethiopia, according to the president`s malaria initiative (PMI) report from 2008–2009, pregnant women accounted for 1.7% of all reported outpatients and 1.7% of inpatient malaria deaths [6].

In stable malaria transmission areas, most infections with *Plasmodium* species in pregnant women remain asymptomatic [7]. Asymptomatic *Plasmodium* species infection (API) is detection of *Plasmodium* species in the blood without any clinical symptoms of malaria. However, there is no standard definition for asymptomatic *Plasmodium* species infection [2, 8]. In addition, according to different scholar’s report, most asymptomatic infections are caused by *P*. *falciparum* and only a few reports are available for other *Plasmodium* species in stable malaria transmission areas [9].

Asymptomatic *Plasmodium* species infection during pregnancy causes placental infection and anaemia [10,11]. Placental infection is caused by sequestration of infected red blood cells (RBCs) in the maternal intervillous spaces of the placenta [12]; and associated with placental inflammation and fibrosis [10,13]. Placental infection also induces a local inflammation and a massive infiltration of immune cells like macrophages, monocytes and lymphocytes and is often described as ‘inflammatory placental malaria [14,15]. This situation disturbs nutrient and air exchanges between the mother and the foetus. This resulted in increased fetal mortality, prematurity, low birth weight, abortion, stillbirths and fetal anaemia [16,17].

Ethiopia has done lots of efforts to improve health status of pregnant women. According to the unpublished data from the Jawi district health office, the antenatal care coverage of the study area was greater than 85%. One of the efforts that has been done at the study area is to prevent pregnant women from infectious diseases like malaria.

Ethiopia has launched malaria elimination program by 2030 and doing lots of efforts. To achieve this goal, currently the country used three major malaria prevention methods: early diagnosis and prompt treatment, vector control measures like use of indoor residual spraying (IRS) and insecticide treated bed nets (ITNs) methods and adoption of surveillance [3]. However, this program has been hampered by asymptomatic cases acting as reservoirs of infection and source of transmission. As a result, asymptomatic infections should be diagnosed and treated as early as possible. Even if, asymptomatic infection during pregnancy causes various effects on the mother and the foetus, they have been missed up by the conventional malaria diagnostic tests because of low parasite density [18]. Moreover, asymptomatic cases, have low health seeking behaviour due to absence of developing disease and hence not treated. As a result, it causes various effects on the mother and foetus like abortion, intrauterine growth retardation, stillbirths, low birth weight, fetal and maternal anaemia and increased maternal and neonatal mortality caused by malaria and malaria related complications [16,17]. Furthermore, asymptomatic pregnant women can act as reservoirs of *Plasmodium* species and transmit the disease to the population [19]. These conditions impaired the control and elimination programs of malaria by the government.

Since asymptomatic *Plasmodium* species infection is common in stable malaria transmission area, this hypothesis holds true at Jawi district. In addition, there is no data that showed the magnitude and associated factors for asymptomatic *Plasmodium* species infection among pregnant women at the study area. Therefore, the aim of this study was to assess the prevalence of asymptomatic *Plasmodium* species infection and associated factors among pregnant women attending antenatal care at Fendeka town health facilities, Jawi district north west Ethiopia.

## Methods and materials

### Study design, period and area

**Fig 1:**
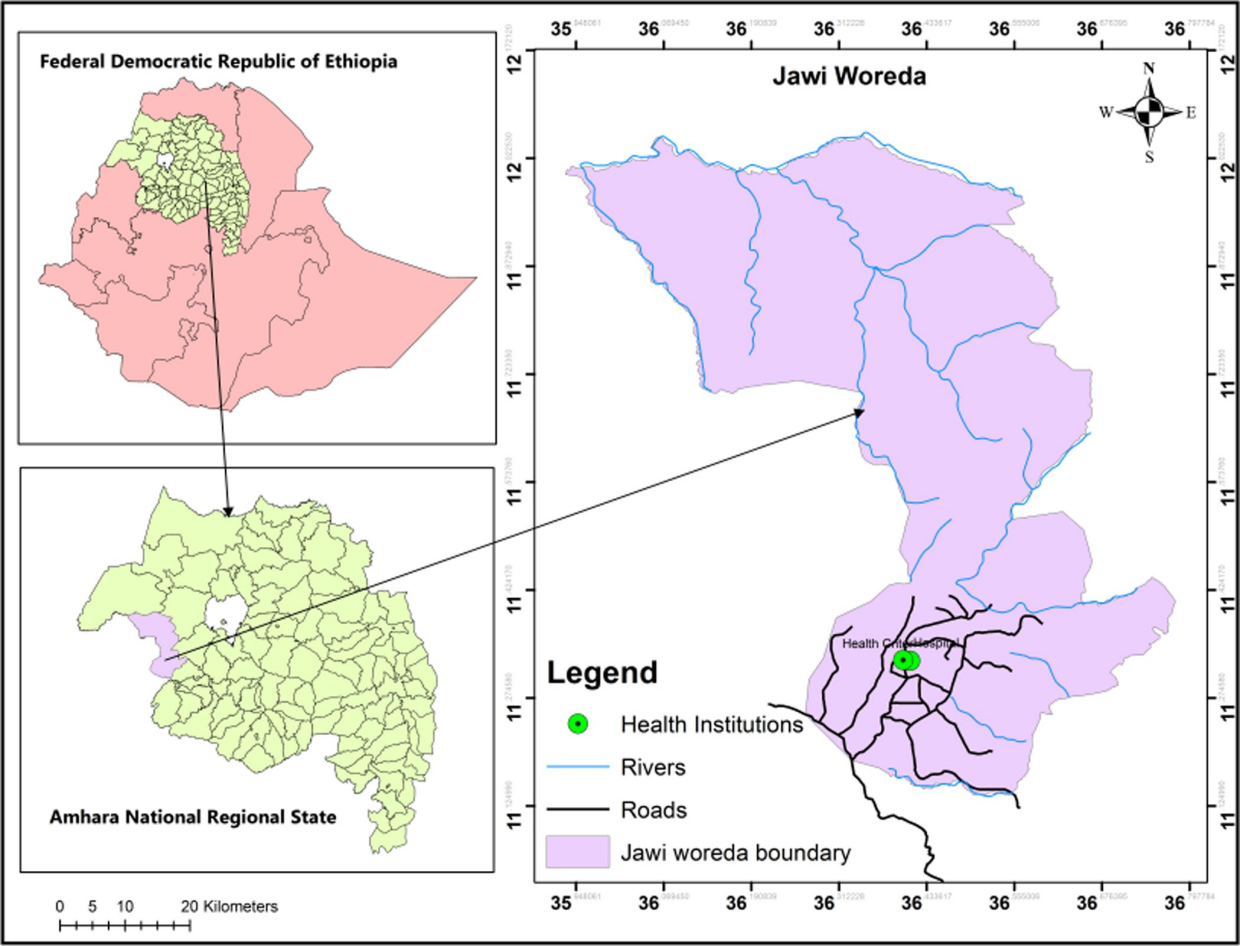
Map of the Federal Republic of Ethiopia and Amhara Regional State showing the study area, Jawi district.

Health facility based cross-sectional study was conducted from February to March 2019 at Fendeka town Jawi district northwest Ethiopia (Fig 1). Jawi district is one of the districts in Amhara region. The mean temperature varies between 16.68 ^0^ c-37.6 ^0^ c and the altitude ranges from 648-1300m above sea level. The number of malaria reported cases in 2018/19 were 24,434 (Jawi district office malaria cases report). Moreover, Tana Beles integrated sugar development project is found at the study area and there is year-round transmission of malaria. According to 2007 national central statistical census report, the total population of the district was 79,090; of whom 41,407 were men and 37,683 women [20]. The district has long summer rain fall (June-September) and a winter dry season (October-May) with mean annual rain fall of 1569.4mm.

### Dependent and independent variables

The dependent variable was asymptomatic *Plasmodium* species infections and the independent variables were: age, education level, occupation, marital status, family size, residence (rural/urban), Gravidity (Primigravidae, secugravidae, multigravidae), gestational age (first trimester, second trimester, third trimester), malaria prevention strategies (ITN ownership and utilization, IRS usage).

### Operational definitions

#### Asymptomatic infection

The detection of *Plasmodium* species in the absence of any clinical symptoms of malaria (usually fever within the past two weeks).

#### Multigravida

A woman who is pregnant for the third time and above.

### Sample size determination and sampling techniques

Sample size was calculated using single population proportion formula; using 9.4% prevalence of asymptomatic *Plasmodium* species infection [21]; and margin error of 3%. Since the total number of pregnant women at the study area were less than 10,000 or total number is 5028, correction formula was used and a total of 331 pregnant women were participated in the study. Then, using proportionate allocation, 166 and 165 participants were involved from Jawi hospital and health centre, respectively. Finally, convenient sampling technique was used at both health facilities to obtain the required sample size. Eighty-three dried blood spot (DBS) samples were also collected at both health facilities using systematic random sampling technique.

### Data collection methods and recruitment of pregnant women

#### Socio-demographic characteristics and associated factors

Usually, pregnant women attend their follow up at the antenatal care clinic (ANC) to check whether the presence or absence of problems and/or anomalies related to pregnancy. As they reached at the ANC, the midwife gave their identity card and examined the status of their pregnancy. After checking their status and if they were normal, the pregnant women were recruited regardless of their appointment period. Then, the midwife requested their willingness to participate in the study. On volunteered pregnant women, socio-demographic characteristics like sex, residence, age, educational level, occupation, marital status, family size, and associated factors like gravidity, gestational age, ownership and utilization of ITNs and use of IRS, previous use of antimalarials during pregnancy and clinical questions like fever, shivering and sweating were collected via a face to face interview by the trained midwives.

#### Blood sample collection

Blood sample was collected from participants pricking at their ring finger for RDTs, thick and thin blood films and DBS using Whatman filter paper. For RDTs, 5μl of blood was added using micropipette to the sample well and 2 drops (60μl) of buffer solution to the buffer well according to the manufacturer’s instruction. For thick and thin blood films preparation, 6 μl and 2 μl blood were used using an automatic pipette and smeared on a slide. In addition, each of the thick and thin films was dried overnight, and the thin films were fixed by dipping in absolute methanol for 10–20 seconds. Then, slides were stained with 3% Giemsa for 30–45 minutes [22]. For DBS, drops of blood were spotted on Whatman filter paper (DBS card) and allow them to dry. After the DBS cards were dried, they were packaged in to each sealed plastic bags with desiccant, it was stored at - 20 0c in the lab. Later, it was transferred to Amhara Public Health Institute for molecular analysis using real-time PCR.

#### Laboratory diagnosis

Parasite detection by CareStart™ Malaria Pf / Pv (HRP2/pLDH) Ag Combo RDTs was done based on manufacturer instructions. Five microliters of capillary blood were added using micropipette to the sample well and 2 drops (60μl) of buffer solution to the buffer well and then after waiting for 20 minutes, the results were reported based on the manufacturers instruction [23]

Thin and thick stained smears were examined under light microscope (Olympus CX-21) with 100x magnification for detection and identification of *Plasmodium* parasites by experienced laboratory professionals working at Jaw health centre and hospital. Thick blood films were considered positive when sexual and asexual forms were detected and considered as negative after observing 200 high power fields without detecting any parasite.

Finally, for molecular analysis, 3-mm punches of the DBS were punched out and placed in to 1.5-ml micro-centrifuge tubes. The punches inside the tube were treated with ATL buffer, proteinase K, AL buffer and 96% ethanol, mixing thoroughly by vortexing, brief centrifuge and incubation with consecutive additions. The mixtures were transferred into QIAamp Mini spin column and with subsequent addition of wash buffer AW1 and AW2, DNA was extracted. The DNA was eluted in 100μl of elution buffer, aliquoted and stored at -20°C until running PCR assay [24]. The PCR assay was run using Agilent Technologies strata gene Mx3005p PCR machine.

*Photo -induced electron transfer PCR (PET-PCR) Primer sequence*.

Original Genus 18sFor, 5’-GGC CTA ACA TGG CTA TGA CG-3’.

Original Genus FAM 18sRev, 5’-agg cgc ata gcg cct ggC TGC CTT CCT TAG ATG TGG TAG CT-3’.

Falciparum For, 5’-ACC CCT CGC CTG GTG TTT TT-3’.

Falciparum Rev, HEX-5’-agg cgg ata ccg cct ggT CGG GCC CCA AAA ATA GGA A-3’.

P. vivax For, 5’-GTA GCC TAA GAA GGC CGT GT-3’.

P. vivax Rev, HEX-5’- agg cgc ata gcg cct ggC CTG GGG GAT GAA TAT CTC TAC AGC ACT GT-3’.

Amplification of *Plasmodium* Genus and *P*. *falciparum* or *P*. *vivax* was performed in a 20μl reaction containing 2X TaqMan Environmental Master Mix buffer, forward and reverse primers for the Genus and *P*. *falciparum* multiplex assays and *P*. *vivax* singleplex assay, and 5μl of DNA samples. Samples were run in duplicate on manually loaded 96-well PCR plates. The reactions performed under the following cycling conditions: initial hot-start at 95oC for 15 minutes, followed by 45 cycles of denaturation at 95 0C for 20 seconds and annealing at 60 0C for 40 seconds for Genus and *P*. *falciparum* multiplex assays.

For *P*. *vivax* single plex assay; initial hot-start at 95oC for 15 minutes, followed by 45 cycles of denaturation at 95 0C for 20 seconds and annealing at 60 0C for 40 seconds and then at 72 0C for 30 seconds. The correct fluorescence channel was selected for each fluorescently labelled primer set and the cycle threshold (CT) values recorded at the end of annealing step. Any sample with a CT value of 40.5 or below was considered positive [24]. The Genus and *P*. *falciparum* multiplex assays were performed for all samples. *P*. *vivax* single-plex assay was performed for Genus positive and *P*. *falciparum* negative samples.

### Data quality control and management

Before the data collection, training was given to the data collectors by the principal investigator how to collect the sample and fill the questionnaire. Socio-demographic characteristics and associated factors were collected by midwives.; while experienced laboratory professionals were involved for RDTs, thin and thick blood film preparation, DBS sample collection and *Plasmodium* species identification. Moreover, the same microscope mark (CX21) was used to minimize the possible instrumental error. The questionnaire were prepared in clear and understandable way. The clarity, understandability, and flow of each question were assessed properly. Data were collected under intensive supervision by the principal investigator (PI). Finally, the discordant results were rechecked by the PI along with other experienced laboratory professionals who did not participate in the initial identification and preparation process.

### Ethical consideration

Ethical approval was obtained from the ethical review board of college of medicine and health sciences, Bahir Dar University. Then, permission letters were also obtained from Amhara public health institute, Zonal health department, district health office and from both health facility managers. After explaining the purpose of the study clearly, written informed consent was obtained from each pregnant woman. The data and sample collected from each study participant were used only for this study and confidentiality was always maintained. Finally, positive results were communicated to the attending midwives and received anti-malaria treatment.

### Data analysis

Data was analysed using SPSS version 20 statistical package. Descriptive statistics was used to assess the prevalence of asymptomatic *Plasmodium* species infection among pregnant women. Univariate logistic regression analyses were performed to assess the association between dependent and independent variables. To filter out the confounding effects, variables with P-value < 0.25 in the univariate logistic regression model were further analysed in multivariate logistic regression analysis. Odds ratios at 95% confidence intervals were calculated and variables with P-value < 0.05 were considered as statistically significant.

## Results

### Socio demographic characteristics and associated factors of the pregnant women

A total of 331 pregnant women were participated and of these 208 (62.8%) were rural dwellers. Most of the participants 111(33.5%) age were between 21–25 years with the median age of 25 years and standard deviation of (SD ± 5.35). Majority 203 (61.3%) of study participants could not read and write and farmers accounted for 174 (52.6%). Most 313 (94.6%) of pregnant women were married and 164 (49.5%) of them had 3–6 family sizes. Among the participants, 154 (46.5%) were multigravida and 151 (45.6%) were at their third trimester. Two hundred fifteen (65%) of the participants possessed ITNs; among these 154 (71.4%) had 1 ITNs at their home; and 197 (91.6%) of them had used by hanging it on their beds. Forty-seven (23.9%) of the participants used ITNs only in rainy season. Among the participants, 253 (76.4%) of them used IRS for the last one year (**Table 1**).

**Table 1:** Socio demographic characteristics and associated factors of the pregnant women attending antenatal care at Fendeka town health facilities from February - March 2019, (N = 331).

Variables		N (%)	*Pos N (%)	COR (95%CI)	P-value
Residence	Rural	208 (62.8)	31 (14.9)	3.42 (1.38–8.44)	0.008**
	Urban	123 (37.2)	6 (4.9)	1	
Age Groups	≤ 20	77 (23.3)	15 (19.5)	2.60 (0.81–8.38)	0.109**
	21–25	111 (33.5)	9 (8.1)	0.95 (0.28–3.25)	0.933
	26–30	96 (29)	9 (9.4)	1.11 (0.32–3.82)	0.866
	≥ 31	47 (14.2)	4 (8.5)	1	
Education level	Unable to read and write	20 3 (61.3)	23 (11.3)	1.41 (0.17–11.40)	0.750
	Able to read and write	47 (14.2)	5 (10.6)	1.31 (0.14–12.39)	0.814
	Grades 1–6	23 (6.9)	2 (8.7)	1.05 (0.09–12.88)	0.971
	Grades 7–10	35 (10.6)	5 (14.3)	1.83 (0.19–17.49)	0.598
	Grades 11–12	11 (3.3)	1 (9.1)	1.10 (0.06–20.01)	0.949
	Diploma and above	12 (3.6)	1 (8.3)	1	
Occupation	Farmer	174 (52.6)	24 (13.8)	1.28 (0.36–4.58)	0.704
	GVT employee	15 (4.5)	1 (6.7)	0.57 (0.05–6.04)	0.642
	Student	6 (1.8)	1 (16.7)	1.60 (0.14–18.72)	0.708
	House wife	109 (32.9)	8 (7.3)	0.63 (0.16–2.57)	0.523
	Self/private employee	27 (8.2)	3 (11.1)	1	
Marital status	Married	313 (94.6)	34 (10.9)	1	
	Single	5 (1.5)	1 (20)	2.05 (0.22–18.89)	0.526
	Divorced	8 (0.3)	1 (12.5)	1.17 (0.14–9.82)	0.883
	Widowed	5 (1.5)	1 (20)	2.05 (0.22–18.89)	0.526
Family size	≤ 2	132 (39.9)	19 (14.4)	2.77 (0.61–12.53)	0.185**
	3–6	164 (49.5)	16 (9.8)	1.78 (0.39–8.14)	0.455
	7–10	35 (10.6)	2 (5.7)	1	
Gravidity	Primigravidae	112 (33.8)	18 (16.1)	2.76 (1.22–6.23)	0.015**
	Secugravidae	65 (19.6)	9 (13.8)	2.31 (0.89–6.00)	0.084**
	Multigravida	154 (46.5)	10 (6.5)	1	
Gestational age	1^st^ trimester	49 (14.8)	5 (10.2)	0.74 (0.26–2.10)	0.577
	2^nd^ trimester	131(39.6)	12 (9.2)	0.66 (0.31–1.41)	0.283
	3^rd^ trimester	151(45.6)	20 (13.2)	1	
ITNs possession	Yes	215 (65)	24 (11.2)	1	
	No	116 (35)	13 (11.2)	1.00 (0.49–2.06)	0.990
ITNs number	1	154 (71.6)	20 (13)	1.19 (0.14–10.06)	0.870
	2	52 (24.3)	3 (5.8)	0.49 (0.05–5.31)	0.557
	3	9 (4.2)	1 (11.1)	1	
Using ITNs by hanging on bed	Yes	197 (91.6)	21 (10.7)	1	
	No	18 (8.4)	3 (16.7)	1.68 (0.45–6.27)	0.443
Reason not used ITNs	Generates heat	11 (61.1)	2 (18.2)	1.33 (0.10–18.19)	0.829
	Looks like suffocated	7 (38.9)	1 (14.3)	1	
IRS use last one year	Yes	253 (76.4)	21 (8.3)	1	
	No	78 (23.6)	16 (20.5)	2.85 (1.40–5.79)	0.004**

GVT: Government N: Number of participants,

*Pos = Positive with RDTs only or microscopy only or PCR only or all the three tests or RDTs and microscopy only.

### Prevalence of asymptomatic *Plasmodium* species infection among pregnant women

The prevalence of asymptomatic *Plasmodium* species infections was 17 (5.1%), 30 (9.1%) and 15 (18.1%) using RDTs, microscopy and real-time PCR, respectively. Of the 15 samples detected by PCR, 8 (53.4%), 5 (33.3%) and 2 (13.3%) were *P*. *falciparum*, *P*. *vivax* and mixed infections, respectively (**Table 2**). This Table depicted that for the detection of asymptomatic *Plasmodium* species infection, PCR was better as compared to RDTs and microscopy.

**Table 2:** Prevalence of *Plasmodium* species infection among pregnant women attending antenatal care at Fendeka town health facilities from February to March, 2019, (N = 331; for PCR, N = 83).

Plasmodium Species	Diagnostic Methods		
	RDTs N = 331	Microscopy N = 331	PCR N = 83
**Pf**	11	17	8
**Pv**	5	11	5
**Mixed**	1	2	2
**Total N (%)**	17 (5.1)	30 (9.1)	15 (18.1)

Pf: *Plasmodium falciparum* Pv: *Plasmodium vivax* Mixed: Pf and Pv.

### Factors associated with asymptomatic *Plasmodium* species infection

Residence, age, gravidity and IRS usage for the last one year had P-value < 0.25 by univariate logistic regression analysis. Moreover, residence, gravidity and IRS usage for the last one year depicted statistically significant association at P- value < 0.05 (**Table 3**).

**Table 3:** Univariate analysis of associated factors for asymptomatic *Plasmodium* species infection among pregnant woman attending antenatal care at Fendeka town health facilities from February - March 2019, (N = 331).

Variables		N (%)	Pos N (%)	COR (95%CI)	P-value
Residence	Rural	208 (62.8)	31 (14.9)	3.42 (1.38–8.44)	0.008**
	Urban	123 (37.2)	6 (4.9)	1	
Age groups	≤ 20	77 (23.3)	15 (19.5)	2.60 (0.81–8.38)	0.109**
	21–25	111 (33.5)	9 (8.1)	0.95 (0.28–3.25)	0.933
	26–30	96 (29)	9 (9.4)	1.11 (0.32–3.82)	0.866
	≥ 31	47 (14.2)	4 (8.5)	1	
Education level	Unable to read and write	20 3 (61.3)	23 (11.3)	1.41 (0.17–11.40)	0.750
	Able to read and write	47 (14.2)	5 (10.6)	1.31 (0.14–12.39)	0.814
	Grades 1–6	23 (6.9)	2 (8.7)	1.05 (0.09–12.88)	0.971
	Grades 7–10	35 (10.6)	5 (14.3)	1.83 (0.19–17.49)	0.598
	Grades 11–12	11 (3.3)	1 (9.1)	1.10 (0.06–20.01)	0.949
	Diploma and above	12 (3.6)	1 (8.3)	1	
Occupation	Farmer	174 (52.6)	24 (13.8)	1.28 (0.36–4.58)	0.704
	GVT employee	15 (4.5)	1 (6.7)	0.57 (0.05–6.04)	0.642
	Student	6 (1.8)	1 (16.7)	1.60 (0.14–18.72)	0.708
	House wife	109 (32.9)	8 (7.3)	0.63 (0.16–2.57)	0.523
	Self/private employee	27 (8.2)	3 (11.1)	1	
Marital status	Married	313 (94.6)	34 (10.9)	1	
	Single	5 (1.5)	1 (20)	2.05 (0.22–18.89)	0.526
	Divorced	8 (0.3)	1 (12.5)	1.17 (0.14–9.82)	0.883
	Widowed	5 (1.5)	1 (20)	2.05 (0.22–18.89)	0.526
Family size	≤ 2	132 (39.9)	19 (14.4)	2.77 (0.61–12.53)	0.185**
	3–6	164 (49.5)	16 (9.8)	1.78 (0.39–8.14)	0.455
	7–10	35 (10.6)	2 (5.7)	1	
Gravidity	Primigravidae	112 (33.8)	18 (16.1)	2.76 (1.22–6.23)	0.015**
	Secugravidae	65 (19.6)	9 (13.8)	2.31 (0.89–6.00)	0.084**
	Multigravida	154 (46.5)	10 (6.5)	1	
Gestational age	1^st^ trimester	49 (14.8)	5 (10.2)	0.74 (0.26–2.10)	0.577
	2^nd^ trimester	131(39.6)	12 (9.2)	0.66 (0.31–1.41)	0.283
	3^rd^ trimester	151(45.6)	20 (13.2)	1	
ITNs possession	Yes	215 (65)	24 (11.2)	1	
	No	116 (35)	13 (11.2)	1.00 (0.49–2.06)	0.990
ITNs number	1	154 (71.6)	20 (13)	1.19 (0.14–10.06)	0.870
	2	52 (24.3)	3 (5.8)	0.49 (0.05–5.31)	0.557
	3	9 (4.2)	1 (11.1)	1	
Using ITNs by hanging on bed	Yes	197 (91.6)	21 (10.7)	1	
	No	18 (8.4)	3 (16.7)	1.68 (0.45–6.27)	0.443
Reason not used ITNs	Generates heat	11 (61.1)	2 (18.2)	1.33 (0.10–18.19)	0.829
	Looks like suffocated	7 (38.9)	1 (14.3)	1	
IRS use last one year	Yes	253 (76.4)	21 (8.3)	1	
	No	78 (23.6)	16 (20.5)	2.85 (1.40–5.79)	0.004**

* Significant at p-value < 0.05.

** Significant at p-value < 0.25 N = Number of participants, Pos = positive, Neg = negative, COR = crude odd ratio, AOR = adjusted odd ratio, CI = confidence interval,

In multivariate logistic regression analysis; residence, gravidity and IRS usage for the last one year had a statistically significant association with asymptomatic *Plasmodium* species infection at P-value < 0.05. This analysis also showed that the odds of being asymptomatic *Plasmodium* species infection was 4.51 times higher among rural dweller pregnant women. The odds of being asymptomatic infection with *Plasmodium* species was 4.51 times higher among primigravida and 3.87 times higher among secugravidae as compared to multigravida. It was found that the odds of being asymptomatic infection with *Plasmodium* species was 3.13 times more likely in pregnant women whose houses did not spray IRS as compared to who have been sprayed IRS for the last one year (**Table 4**).

**Table 4:** Multivariate analysis of associated factors for asymptomatic *Plasmodium* infection among pregnant woman attending antenatal care at Fendeka town health facilities from February - March 2019, (N = 331).

Variables		N (%)	Pos N (%)	AOR (95% CI)	P-value
Residence	Rural	208 (62.8)	31 (14.9)	4.51 (1.72–11.84)	0.002*
	Urban	123 (37.2)	6 (4.9)	1	-
Age groups	≤ 20	77 (23.3)	15 (19.5)	0.65 (0.12–3.47)	0.611
	21–25	111 (33.5)	9 (8.1)	0.41 (0.09–1.96)	0.264
	26–30	96 (29)	9 (9.4)	0.83 (0.21–3.28)	0.790
	≥ 31	47 (14.2)	4 (8.5)	1	-
Education level	Unable to read and write	203 (61.3)	23 (11.3)	-	-
	Able to read and write	47 (14.2)	5 (10.6)	-	-
	Grades 1–6	23 (6.9)	2 (8.7)	-	-
	Grades 7–10	35 (10.6)	5 (14.3)	-	-
	Grades 11–12	11 (3.3)	1 (9.1)	-	-
	Diploma and above	12 (3.6)	1 (8.3)	-	-
Occupation	Farmer	174 (52.6)	24 (13.8)	-	-
	GVT employee	15 (4.5)	1 (6.7)	-	-
	Student	6 (1.8)	1 (16.7)	-	-
	House wife	109 (32.9)	8 (7.3)	-	-
	Self/private employee	27 (8.2)	3 (11.1)	-	-
Marital status	Married	313 (94.6)	34 (10.9)	-	-
	Single	5 (1.5)	1 (20)	-	-
	Divorced	8 (0.3)	1 (12.5)	-	-
	Widowed	5 (1.5)	1 (20)	-	-
Family size	≤ 2	132 (39.9)	19 (14.4)	-	-
	3–6	164 (49.5)	16 (9.8)	-	-
	7–10	35 (10.6)	2 (5.7)	-	-
Gravidity	Primigravidae	112 (33.8)	18 (16.1)	4.51 (1.27–16.03)	0.020*
	Secugravidae	65 (19.6)	9 (13.8)	3.87(1.16–12.93)	0.028*
	Multigravidae	154 (46.5)	10 (6.5)	1	-
Gestational age	1^st^ trimester	49 (14.8)	5 (10.2)	-	-
	2^nd^ trimester	131 (39.6)	12 (9.2)	-	-
	3^rd^ trimester	151 (45.6)	20 (13.2)	-	-
ITNs possession	Yes	215 (65)	24 (11.2)	-	-
	No	116 (35)	13 (11.2)	-	-
ITNs number	1	154 (71.6)	20 (13)	-	-
	2	52 (24.3)	3 (5.8)	-	-
	3	9 (4.2)	1 (11.1)	-	-
Using ITNs by hanging on bed	Yes	197 (91.6)	21 (10.7)	-	-
	No	18 (8.4)	3 (16.7)	-	-
Reason not used ITNs	Generates heat	11 (61.1)	2 (18.2)	-	-
	Looks like suffocated	7 (38.9)	1 (14.3)	-	-
IRS use last one year	Yes	253 (76.4)	21 (8.3)	1	-
	No	78 (23.6)	16 (20.5)	3.13 (1.47–6.66)	0.003*

* Significant at p-value < 0.05.

** Significant at p-value < 0.25 N = Number of participants, Pos = positive, Neg = negative, COR = crude odd ratio, AOR = adjusted odd ratio, CI = confidence interval,

## Discussion

Asymptomatic *Plasmodium* species infection could be an obstacle in malaria elimination program due to the low health seeking behaviours of infected individuals and absence of standardized diagnostic tests. As a result, they become reservoirs for sustainable malaria transmission [9]. Especially, during pregnancy API causes many problems in pregnant women and the developing foetus and new-borns.

Rapid diagnostic tests detected and identified 5.1% of the APIs. This result is in line with study done in Nigeria 4.8% [25]. But, it is lower than reports from Burkina Faso 30% [12], Congo 27.4% [26], Nigeria 13.1% [27] and south Ethiopia 9.7% [21]. These differences might be due to data collection period [12, 21], and other studies were community based [21].

Microscopy detected and identified 9.1% of asymptomatic *Plasmodium* species infections among pregnant women. This result is consistent with studies done in south Ethiopia 9.1% [21], Congo 7% [28] and Nigeria 9.2% [27]. However, this is lower than studies done in Nigeria 58.4% [29], and other reports from Nigeria 48% and 22.7% [30, 31], Burkina Faso 24% [12] and Congo 21.6% [26]. In contrast, this microscopy result is higher than other study done in Nigeria 3.1% [25]. These discrepancies might be due to data collection period and seasonality [12, 29, 31], very low land area (230 meter above sea level) in our study area [29].

Real-time PCR detected and identified 18.1% of the asymptomatic *Plasmodium* species infection. It is consistent with the study done in Congo 19% [28]. On the other hand, this is lower than study done in Congo 29.5% [26]. These variations might be due to partial immunity within the community.

Sixty-two percent, 33% and 5% of the *Plasmodium* species were identified as *P*. *falciparum*, *P*. *vivax* and mixed infections, respectively. This result is in line with MoH report that states 60% and 40% of the *Plasmodium* species in Ethiopia are *P*. *falciparum* and *P*. *vivax*, respectively [4]; and with the study done in south Ethiopia [21].

Factors associated with APIs depicted that residence, gravidity and IRS use for the last one year were statistically significant with APIs. Rural dwellers were 4.51 times more likely exposed to *Plasmodium* species infection compared to urban dwellers. This could be explained by the fact that *Plasmodium* species infection is usually higher in the rural environment where *Anopheles mosquito* breeding and malaria transmission is more intense [32, 33].

Women in their first pregnancy were 4.51 times and second pregnancies were 3.87 times more likely developed asymptomatic *Plasmodium* species infection than multigravida. This finding agrees with the studies done in south Ethiopia [21], Nigeria [29, 31] and Congo [26]. In addition, this association agrees with findings of similar studies from sub-Saharan African countries where the prevalence of asymptomatic *Plasmodium* species infection was significantly higher in primigravida than multigravida [34,35].

Pregnant women whose house did not spray IRS for the last one year were 3.13 times more likely develop APIs as compared to those pregnant women whose house sprayed IRS. This result is inconsistent with study finding in south Ethiopia that IRS usage was inversely associated with prevalence of APIs [21]. This difference might be due to improper use of IRS in south Ethiopia [21].

### Limitation of the study

The limitation of this study was unable to diagnose all participants by real-time PCR due to limited resources and the data were collected during the dry season which do not address the wet season. Moreover, we did not take sample from placenta.

## Conclusions

This study showed significant number of asymptomatic *Plasmodium* species infections among pregnant women. Pregnant women who reside in the rural area, primigravida, secugravida and those did not use IRS for the last one year were at higher risk of API infection.

## Supporting information

S1 File (SAV)

## References

[1] Conroy AL, McDonald CR, Kain KC. Malaria in pregnancy: diagnosing infection and identifying fetal risk. Expert Rev Anti-Infect Therapy 2012; 10(11): 1331–1342. 10.1586/eri.12.123 23241190

[2] WHO. (2017) World Health Organization; Factsheet on the World Malaria Report, [Accessed 9 Dec. 2018].

[3] Ministry of Health of Ethiopia (MOH). National Strategic Plan for Malaria Prevention, Control and Elimination in Ethiopia: 2014–2020: Addis Ababa.2014.

[4] Ministry of Health of Ethiopia (MOH): National Malaria Guidelines, 3rd Ed, FMOH, Ethiopia, Addis Ababa, 2012.

[5] Gemeda AW, Wakgari D, Ahmed A, Lindtjørn B. Shifting to *Plasmodium vivax* dominance at highlands of Ethiopia following the countrywide intensive use of artemether-lumefantrine throughout endemic areas since 2005: Can we neglect the role of climate and relapse cases? Artig Puplicad Anaise 2012; 6(7): 187.

[6] PMI. President’s malaria initiative Ethiopia, Malaria Operational Plan FY 2018.

[7] Tagbor H, Bruce J, Browne E, Greenwood B, Chandramohan D. Malaria in pregnancy in an area of stable and intense transmission: is it asymptomatic? Trop Medicin Intern Health 2008; 13(8): 1016–1021. 10.1111/j.1365-3156.2008.02111.x 18631316

[8] Lindblade KA, Steinhardt L, Samuels A, Kachur SP, Slutsker L. The silent threat: asymptomatic parasitemia and malaria transmission. Expert Rev Anti-Infect Therapy 2013; 11(6): 623–639. 10.1586/eri.13.45 23750733

[9] Harris I, Sharrock WW, Bain LM, Gray KA, Bobogare A, Boaz L, et al A large proportion of asymptomatic Plasmodium infections with low and sub-microscopic parasite densities in the low transmission setting of Temotu Province, Solomon Islands: challenges for malaria diagnostics in an elimination setting. Malaria J 2010; 9(1): 254. 10.1186/1475-2875-9-254 PMC2944341 20822506

[10] Mayengue PI, Rieth H, Khattab A, Issifou S, Kremsne PG, Klinkert MQ, et al Submicroscopic *Plasmodium falciparum* infections and multiplicity of infection in matched peripheral, placental and umbilical cord blood samples from Gabonese women. Trop Medicin Intern Health 2004; 9(9): 949–958. 10.1111/j.1365-3156.2004.01294.x 15361107

[11] Mockenhaupt FP, Bedu-Addo G, Junge C, Hommerich L, Eggelte TA, Bienzle U. Markers of sulfadoxine-pyrimethamine-resistant Plasmodium falciparum in placenta and circulation of pregnant women. Anti-microb agent’s chemotherapy 2007; 51(1): 332–334. 10.1128/AAC.00856-06 PMC1797640 17088491

[12] Douamba Z, Bisseye C, Djigma FW, Compaore TR, Bazie V, Pietra V, et al Asymptomatic malaria correlates with anemia in pregnant women at Ouagadougou, Burkina Faso. Bio Med Res Intern 2012: 2. 10.1155/2012/198317 PMC3511849 23226937

[13] Cottrell G, Moussiliou A, Luty AJ, Cot M, Fievet N, Massougbodj A, et al Sub-microscopic *Plasmodium falciparum* infections are associated with maternal anaemia, premature births, and low birth weight. Clin Infect Dis 2015; 60(10): 1481–1488. 10.1093/cid/civ122 25694651

[14] Ordi J, Menendez C, Ismail MR, Ventura PJ, Palacín A, Kahigwa E, et al Placental malaria is associated with cell-mediated inflammatory responses with selective absence of natural killer cells. J Infect Dis 2001; 183(7): 1100–1107. 10.1086/319295 11237836

[15] Nosten F, Rogerson SJ, Beeson JG, McGready R, Mutabingwa TK, Brabin B. Malaria in pregnancy and the endemicity spectrum: what can we learn? Trends Parasitol 2004; 20(9):425–432. 10.1016/j.pt.2004.06.007 15324733

[16] Leopardi O, Naughten W, Salvia L, Colecchia M, Matteelli A, Zucchi A, et al Malaric placentas: a quantitative study and clinico-pathological correlations. Pathol Res Pract 1996; 192(9): 892–898. 10.1016/S0344-0338(96)80068-9 8950755

[17] Menendez C, Ordi J, Ismail MR, Ventura PJ, Aponte JJ, Kahigwa E, et al The impact of placental malaria on gestational age and birth weight. J Infect Dis 2000; 181(5): 1740–1745. 10.1086/315449 10823776

[18] Bottius E, Guanzirolli A, Trape JF, Rogier C, Konate L, Druilhe P. Malaria: even more chronic in nature than previously thought; evidence for sub patent parasitaemia detectable by the polymerase chain reaction. Transact Royal Societ Trop Medicin Hyg 1996; 90(1): 15–19. 10.1016/s0035-9203(96)90463-0 8730301

[19] Gruninger H, Hamed K. Transitioning from malaria control to elimination: the vital role of ACTs. Trends Parasitol 2013; 29(2): 60–64. 10.1016/j.pt.2012.11.002 23228225

[20] Federal Democratic Republic of Ethiopia. Population census commission: Summary and statistical report of the 2007 population and housing census: Population size by age and sex. Addis Ababa.

[21] Nega D, Dana D, Tefera T, Eshetu T, (2015). Prevalence and predictors of asymptomatic malaria parasitemia among pregnant women in the rural surroundings of Arbaminch Town, South Ethiopia. PLoS One 2015; 10(4) 10.1371/journal.pone.0123630 PMC4388389 25849587

[22] Malaria microscopy quality manual, WHO 2016 Version-2.

[23] http://www. accessbio. net/eng/products/products 01_02.asp.

[24] Lucchi NW, Narayanan J, Karell MA, Xayavong M, Kariuki S, DaSilva AJ, et al Molecular diagnosis of malaria by photo-induced electron transfer fluorogenic primers: PET-PCR. PloS One 2013; 8(2) 10.1371/journal.pone.0056677 PMC3577666 23437209

[25] Isah AY, Amanabo MA, Ekele BA, (2011). Prevalence of malaria parasitemia amongst asymptomatic pregnant women attending a Nigerian teaching hospital. Annals African Medicin 2011; 10(2): 171–173. 10.4103/1596-3519.82070 21691026

[26] Matangila JR, Lufuluabo J, Ibalanky AL, da Luz RAI, Lutumba P, Van Geertruyden JP. Asymptomatic Plasmodium falciparum infection is associated with anaemia in pregnancy and can be more cost-effectively detected by rapid diagnostic test than by microscopy in Kinshasa, Democratic Republic of the Congo. Malaria J 2014; 13(1): 132. 10.1186/1475-2875-13-132 PMC3976674 24690179

[27] Obebe OO, Falohun OO, Olajuyigbe OO, Lawani MA, Ajayi OA. Impact of asymptomatic Plasmodium falciparum on haematological parameters of pregnant women at first antenatal visit in South-western Nigeria. Tanzania J Health Res 2018; 20(2): 4.

[28] Francine N, Damien B, Anna F, Michael K, Christevy VJ, Felix KK. Characterization of asymptomatic *Plasmodium falciparum* infection and its risk factors in pregnant women from the Republic of Congo. Acta Trop 2016 153: 111–115. 10.1016/j.actatropica.2015.10.009 26477849

[29] Nwagha UI, Ugwu VO, Nwagha TU, Anyaehie BU. Asymptomatic Plasmodium parasitaemia in pregnant Nigerian women: almost a decade after Roll Back Malaria. Transact Royal Societ Trop Medicin Hyg 2009; 103(1): 16–20. 10.1016/j.trstmh.2008.07.016 18783809

[30] Balogun ST, Adeyanju AA, Adedeji AA, Fehintola FA. Predictors of asymptomatic malaria in pregnancy. Nigerian J Physiol Scien 2011; 26: 179–183. 22547188

[31] Emiasegen SE, Giwa FJ, Ajumobi O, Ajayi IO, Ahmed SA, Olayinka AT. Asymptomatic Plasmodium falciparum parasitaemia among pregnant women: a health facility-based survey in Nassarawa-Eggon, Nigeria. Malaria World J 2017; 15(24): 25–34. PMC8415059 34532231

[32] Robert V, Macintyre K, Keating J, Trape JF, Duchemin JB, Warren M, et al Malaria transmission in urban sub-Saharan Africa. American J Trop Medicin Hyg 2003; 68(2): 169–176. 12641407

[33] Byrne N. Urban malaria risk in sub-Saharan Africa: where is the evidence? Travel Medicin Infect Dis 2007; 5(2): 135–137. 10.1016/j.tmaid.2006.04.003 17298922

[34] Geertruyden JPV, Ntakirutimana D, Erhart A, Rwagacondo C, Kabano A, DAlessandro U. Malaria infection among pregnant women attending antenatal clinics in six Rwandan districts. Trop Medicin Intern Health 2005; 10(7): 681–688. 10.1111/j.1365-3156.2005.01431.x 15960707

[35] Okafor IM, Mbah M, Usanga EA. The impact of anaemia and malaria parasite infection in pregnant women, Nigerian perspective. J Dent Med Scien 2012; 1(1): 34–8.

